# Identification of Potential Biomarkers for Pan-Cancer Diagnosis and Prognosis Through the Integration of Large-Scale Transcriptomic Data

**DOI:** 10.3389/fphar.2022.870660

**Published:** 2022-05-23

**Authors:** Lin Zhu, Yu Miao, Feng Xi, Pingping Jiang, Liang Xiao, Xin Jin, Mingyan Fang

**Affiliations:** ^1^ BGI College and Henan Institute of Medical and Pharmaceutical Sciences, Zhengzhou University, Zhengzhou, China; ^2^ BGI-Shenzhen, Shenzhen, China; ^3^ College of Life Sciences, University of Chinese Academy of Sciences, Beijing, China

**Keywords:** biomarkers, pan-cancer, transcriptome analyses, diagnosis, therapeutic

## Abstract

Cancer is one of the leading causes of death worldwide, bringing a significant burden to human health and society. Accurate cancer diagnosis and biomarkers that can be used as robust therapeutic targets are of great importance as they facilitate early and effective therapies. Shared etiology among cancers suggests the existence of pan-cancer biomarkers, performance of which could benefit from the large sample size and the heterogeneity of the studied patients. In this study, we conducted a systematic RNA-seq study of 9,213 tumors and 723 para-cancerous tissue samples of 28 solid tumors from the Cancer Genome Atlas (TCGA) database, and 7,008 normal tissue samples from the Genotype-Tissue Expression (GTEx) database. By differential gene expression analysis, we identified 214 up-regulated and 186 downregulated differentially expressed genes (DEGs) in more than 80% of the studied tumors, respectively, and obtained 20 highly linked up- and downregulated hub genes from them. These markers have rarely been reported in multiple tumors simultaneously. We further constructed pan-cancer diagnostic models to classify tumors and para-cancerous tissues using 10 up-regulated hub genes with an AUC of 0.894. Survival analysis revealed that these hub genes were significantly associated with the overall survival of cancer patients. In addition, drug sensitivity predictions for these hub genes in a variety of tumors obtained several broad-spectrum anti-cancer drugs targeting pan-cancer. Furthermore, we predicted immunotherapy sensitivity for cancers based on tumor mutational burden (TMB) and the expression of immune checkpoint genes (ICGs), providing a theoretical basis for the treatment of tumors. In summary, we identified a set of biomarkers that were differentially expressed in multiple types of cancers, and these biomarkers can be potentially used for diagnosis and used as therapeutic targets.

## Introduction

Cancer is a serious threat to human life and health and is an important public health problem worldwide. According to the World Health Organization 2021, cancer is the first or second leading cause of human death (Siegel et al., 2021). Cancer arises from genetic mutations and dysregulation of transcriptional processes (Huntsman and Ladanyi, 2018), and its development is influenced by a variety of factors, including cancer cell development, differentiation, and epigenetic regulation (Graham and Sottoriva, 2017).

Early detection and accurate diagnosis of cancer based on robust biomarkers are of great value as they dramatically improve the therapeutic outcome (Sharma, 2018). Reduced costs through molecularly targeted therapies and improved accessibility of early and accurate diagnosis for cancer patients can ultimately lead to better clinical decision-making and additional possibilities for the precision treatment of tumors. Many studies have identified markers that can be used for cancer diagnosis, however, most of them have focused on diagnostic markers for a single type of cancer.

In recent years, pan‐cancer analysis brought us to a new level of cancer research, which overcomes the limitation in the sample size of single-cancer studies, and it is powerful for studying a highly heterogeneous disease like cancer. These pan-cancer studies have led to an increasing understanding of the complexity and heterogeneity of tumors. For example, in human pan-cancer studies, overexpression of BRCA1-associated protein (BRAP) was associated with poor prognosis and immune infiltration in a variety of cancers (Ju et al., 2020a). *PINK1* was down-regulated in most tumors and may play a protective role in cancer patients (Zhu et al., 2020). *NFE2L2* was positively associated with immune infiltration in pan-cancer (Ju et al., 2020b). Pan-cancer research has allowed us to understand that the same cancer may be very different at the molecular level (Hu et al., 2017), while diverse cancers may share the same molecular profile (Comprehensive genomic cha, 2008). Thereby deepening the pan-cancer level studies with a large sample size will hopefully discover new biomarkers which can be used to develop new cancer treatment strategies.

Currently, the TCGA (Weinstein et al., 2013) project provides sufficient transcriptome-level data to allow a systematic analysis of a wide range of cancers. However, early diagnostic studies of tumors also require a large amount of transcriptomic data of matched normal tissues to conduct the differential analysis. Although the TCGA has matched some para-cancerous tissues transcriptome datasets, the sample size is limited. GTEx (Human genomics. The Genot, 2015; Melé et al., 2015) samples can serve as alternative high-quality matched tissue controls, which provides an excellent opportunity to elucidate the transcriptional variation between normal and tumor tissues and the underlying genetic basis of the normal-to-tumor transition.

There are different treatment modalities for each tumor at present, while fewer broad-spectrum anti-cancer drugs are available, and it is urgent to find more broad-spectrum anti-cancer drugs. In addition, except for traditional treatment modalities like chemotherapy, immunotherapy represented by immune-checkpoint inhibitors (ICIs) has significantly improved the survival status of cancer patients, which is now changing the way of cancer treatment. However, the response to ICIs varies dramatically among patients with different malignancies. Therefore, screening the appropriate immunotherapy population before treatment is crucial to achieving precise treatment (Wang et al., 2019a).

In this study, we identified DEGs that are consistently expressed differentially in different cancers by performing differential gene expression analysis on transcriptomic data from TCGA and GTEx databases. A pan-cancer diagnostic model was further constructed to classify pan-cancers and normal samples with good performance. We also investigated the feasibility of using hub genes for prognostic assessment and drug sensitivity prediction. This study identified a set of candidate pan-cancer biomarkers, brought new insight into the etiology of tumors, and potentially provided new therapeutic targets for some cancers.

## Materials and Methods

### Data Acquisition

The transcriptome data of tumor samples and part of the matched control samples were obtained from the TCGA, including 9,213 tumor tissue samples and 723 para-cancerous tissue samples. In addition, we matched 7,008 tissue samples from GTEx (https://gtexportal.org/home/) database to obtain the transcriptomic data of normal tissues. The related normalized mRNA data and clinical data were downloaded from the UCSC Xena database (https://xena.ucsc.edu/) and used for subsequent differential gene expression analysis.

### Differential Expression Gene Analysis

The DEGs were identified using the DESeq2 R package (1.30.1). Genes were defined as differentially expressed by thresholds of adjusted *p*-value < 0.05 and absolute log2 fold change (FC) > 1.0. When readings for genes are not detected in half or more of the samples, these genes will be filtered out by quality control.

### Pathway Enrichment Analysis

Functional enrichment analysis based on the Kyoto Encyclopaedia of Genes and Genomes (KEGG) database (Kanehisa and Goto, 2000) was performed using Kobas3.0 (Bu et al., 2021). The top 30 items of the KEGG pathway enrichment analysis were sorted and presented as bar charts, which were plotted with the ggplot2 R package based on the *p* values of the statistical software R (version 4.0.4). *p* < 0.05 was considered to be statistically significant.

### Protein-Protein Interaction Network Construction

The interaction between the DEGs was identified by the Search Tool for the Retrieval of Interacting Genes/Proteins database (STRING v10.5) (https://string-db.org/) and visualized using Cytoscape software (Shannon et al., 2003). The top 10 genes with the highest degree of connectivity were then selected as the target hub genes by using the Cytoscape plugin cytoHubba. The criteria of the protein-protein interaction (PPI) network included a confidence score ≥ 0.4.

### Survival Analysis

Patient survival and related analysis were performed on Gene Expression Profiling Interactive Analysis 2 (Tang et al., 2019) (GEPIA2, http://gepia2.cancer-pku.cn), which is a web server built for analyzing the RNA expression data of tumors and normal samples from the TCGA and the GTEx projects, using a standard processing pipeline. GEPIA2 was also used for generating survival heatmaps of hub genes.

### Drug Response Prediction

Drug response prediction was carried out using Gene Set Cancer Analysis (GSCA, http://bioinfo.life.hust.edu.cn/GSCA/) with 20 up- and downregulated hub genes as input, respectively. GSCA is an integrated database for genomic and immunogenomic gene set cancer analysis, which predicts the drug response based on the calculated correlation between mRNA expression and drug IC50 (Liu et al., 2018). The IC50s of more than 700 small molecule drugs in over 1,000 cell lines were obtained from the database of Genomics of Drug Sensitivity in Cancer (GDSC) and the mRNA expression of over 10,000 samples corresponding to more than 30 cancer types from the database of the Genomics of Therapeutics Response Portal (CTRP), respectively.

### Construction of the Classification Model

Classification of tumors and para-cancerous samples was performed using LASSO regression analysis. When the ratio of the number of tumors and para-cancerous samples is severely imbalanced, the accuracy of classification may be affected. Therefore, when the number of tumor samples exceeded 1.5 times the number of para-cancerous tissue samples, we randomly sampled the tumor samples according to the minimum sample size to make sure that the sample sizes of the two groups were approximately equal and repeated 10 times to verify the accuracy of classification.

## Result

### Characteristics of the Studied Samples

**FIGURE 1:**
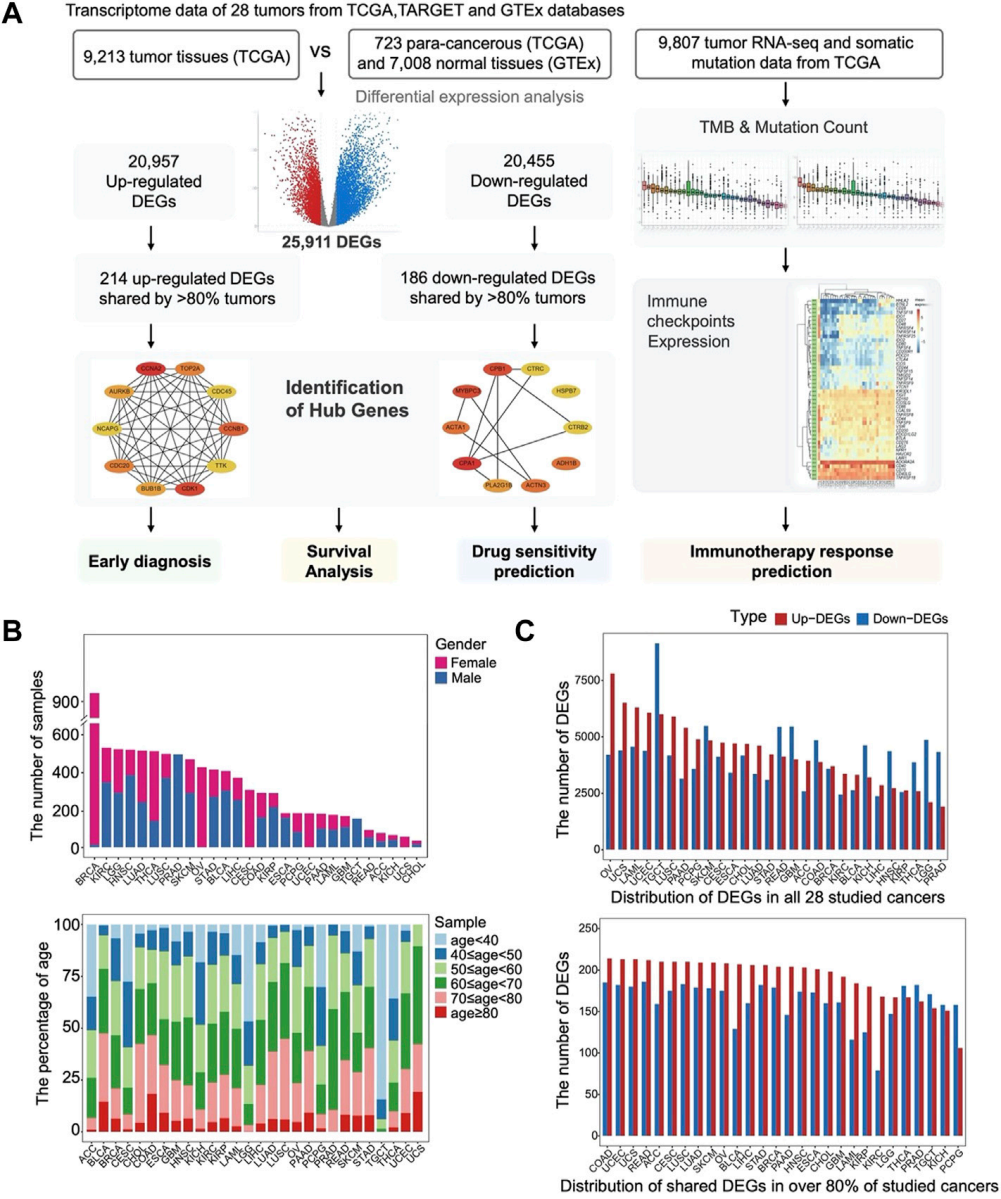
The landscape of distribution of DEGs among all tumors. **(A)** Workflow depicting a collection of TCGA, GTEx datasets, and processing of bioinformatic analysis for RNA-seq of pan-cancer. **(B)** Age and gender distribution of the 28 tumor samples. **(C)** Distribution of DEGs in all 28 studied cancers and distribution of shared DEGs in over 80% of studied cancers.

Transcriptome data were obtained from TCGA and GTEx databases and included 9,213 tumor tissue samples, 723 para-cancerous tissue samples, and GTEx 7,008 normal tissue samples. The tumor types of the samples used in this study, as well as their corresponding sample sizes, and AJCC pathologic stages, are described in Table 1 and Supplementary Figure S1. We set out to identify genes that are consistently differentially expressed in different tumors as a basis for early tumor diagnosis and the prediction of drug targets. The analyses performed in the study were shown as a flowchart diagram in Figure 1A.

**TABLE 1:** 28 cancer types from TCGA and corresponding normal tissue samples from GTEx used for gene expression profiling.

Abbreviations	Full Name	GTEx tissue type	No. of samples		
			TCGA-tumor	TCGA-Normal	GTEX
ACC	Adrenocortical carcinoma	Adrenal gland	77	0	128
BLCA	Bladder urothelial carcinoma	Bladder	407	19	9
BRCA	Breast invasive carcinoma	Breast	1,099	113	179
CESC	Cervical squamous cell carcinoma and endocervical adenocarcinoma	Cervix uteri	306	3	10
CHOL	Cholangiocarcinoma	Liver	36	9	110
COAD	Colon adenocarcinoma	Colon	290	41	308
ESCA	Esophageal carcinoma	Esophagus	182	13	655
GBM	Glioblastoma multiforme	Brain	166	5	1,152
HNSC	Head and neck squamous cell carcinoma	Salivary gland	520	44	55
KICH	Kidney chromophobe	Kidney	66	25	28
KIRC	Kidney renal clear cell carcinoma	Kidney	531	72	28
KIRP	Kidney renal papillary cell carcinoma	Kidney	289	32	28
LAML	Acute myeloid leukemia	Blood	173	0	337
LGG	Brain lower grade glioma	Brain	523	0	1,152
LIHC	Liver hepatocellular carcinoma	Liver	371	50	110
LUAD	Lung adenocarcinoma	Lung	515	59	288
LUSC	Lung squamous cell carcinoma	Lung	498	50	288
OV	Ovarian serous cystadenocarcinoma	Ovary	427	0	88
PAAD	Pancreatic adenocarcinoma	Pancreas	179	4	167
PCPG	Pheochromocytoma and paraganglioma	Adrenal gland	182	3	128
PRAD	Prostate adenocarcinoma	Prostate	496	52	100
READ	Rectum adenocarcinoma	Colon	93	10	308
SKCM	Skin cutaneous melanoma	Skin	469	1	557
STAD	Stomach adenocarcinoma	Stomach	414	36	175
TGCT	Testicular germ cell tumors	Testis	154	0	165
THCA	Thyroid carcinoma	Thyroid	512	59	279
UCEC	Corpus endometrial carcinoma	Uterus	181	23	88
UCS	Uterine carcinosarcoma	Uterus	57	0	88

Overall, the most abundant malignancies in men are PRAD, HNSC, LUSC, and KIRC, while in women, the most common cancers are BRCA, OV, THCA, CESC, and LUAD (Figure 1B). The sample sizes of the studied tumors are approximately consistent with the incidence of the diseases (Siegel et al., 2021). For most malignant tumors, the frequencies of the studied samples increase with age (Figure 1B), which are consistent with the statistic that malignant tumor incidence is low among young people under 40 years of age, but increases rapidly after 40 years of age, and peaks at over 60 years of age. Notably, the age distribution is not uniform across different malignant tumors. The malignant tumors with a younger age of onset are TGCT, LGG, ACC, and THCA, while the other tumors are mainly from patients with age >50. Overall, the data from the study were selected to be representative in terms of sample size and its distribution among different tumors, and the findings can reflect the tumor and pan-cancer related patterns to a certain extent.

### Identification of DEGs in Each Tumor and Shared DEGs in More Than 80% of Tumors

We first explored the unique and common gene expression dysregulation in different tumors by identifying the DEGs in each tumor. We identified a total of 25,911 DEGs in 28 tumors. A histogram shows the occurrence of total DEGs in different tumors (Figure 1C). The number of DEGs varies in different cancers, and the mean and median number of DEGs in each tumor are 8,440 and 8,300, respectively.

We further looked for shared DEGs that expressed differentially in most types of cancers. Among them, a total of 12 genes were found to be differentially expressed in all 28 tumors, of which 6 genes (*ASPM*, *KIF4A*, *NEIL3*, *DTL*, *UBE2C*, and *UBE2SP2*) were upregulated and 2 genes (*PLIN4* and *ADH1B*) were downregulated in expression. In addition, four genes were differentially expressed in opposite directions among 28 tumors, *ABCA9* was upregulated in LAML and down-regulated in the remaining 27 tumors, *NPPA* was upregulated in GBM and LGG and down-regulated in the remaining 26 tumors, while *PBK* and *H2AC11* were down-regulated in TGCT and up-regulated in the rest 27 tumors.

Most of these DEGs have been reported to have potential as diagnostic and prognostic biomarkers in a specific tumor or class of tumors (Xu et al., 2019; Zhao et al., 2021; Luo et al., 2022), while a few have been reported to have such potential in no less than 10 tumors (Jiang et al., 2021; Pan et al., 2021). For example, ASPM (Assembly Factor for Spindle Microtubules) facilitates the homologous recombination-mediated repair of DNA double-strand break (Xu et al., 2021), is essential for mitotic spindle function during cell replication, and plays a pivotal role in the invasiveness of bladder cancer and serves as a potential prognostic biomarker for them (Xu et al., 2019). The UBE2C (Ubiquitin Conjugating Enzyme E2 C) is a conjugating enzyme, that plays a crucial role in cancer progression and its upregulation has been found in various cancers, a recent study demonstrated that overexpression of UBE2C was associated with TMB, microsatellite instability, immune cell infiltration, and diverse drug sensitivities (Jiang et al., 2021).

In addition, there are 47 genes differentially expressed in 27 types of cancers, while 677 are differentially expressed in >80% of tumor types (Supplementary Tables S1, S2). The distribution of genes that were consistently up- or downregulated in more than 80% of tumors was shown in Figure 1C.

### Functional Annotation of Shared DEGs

**FIGURE 2:**
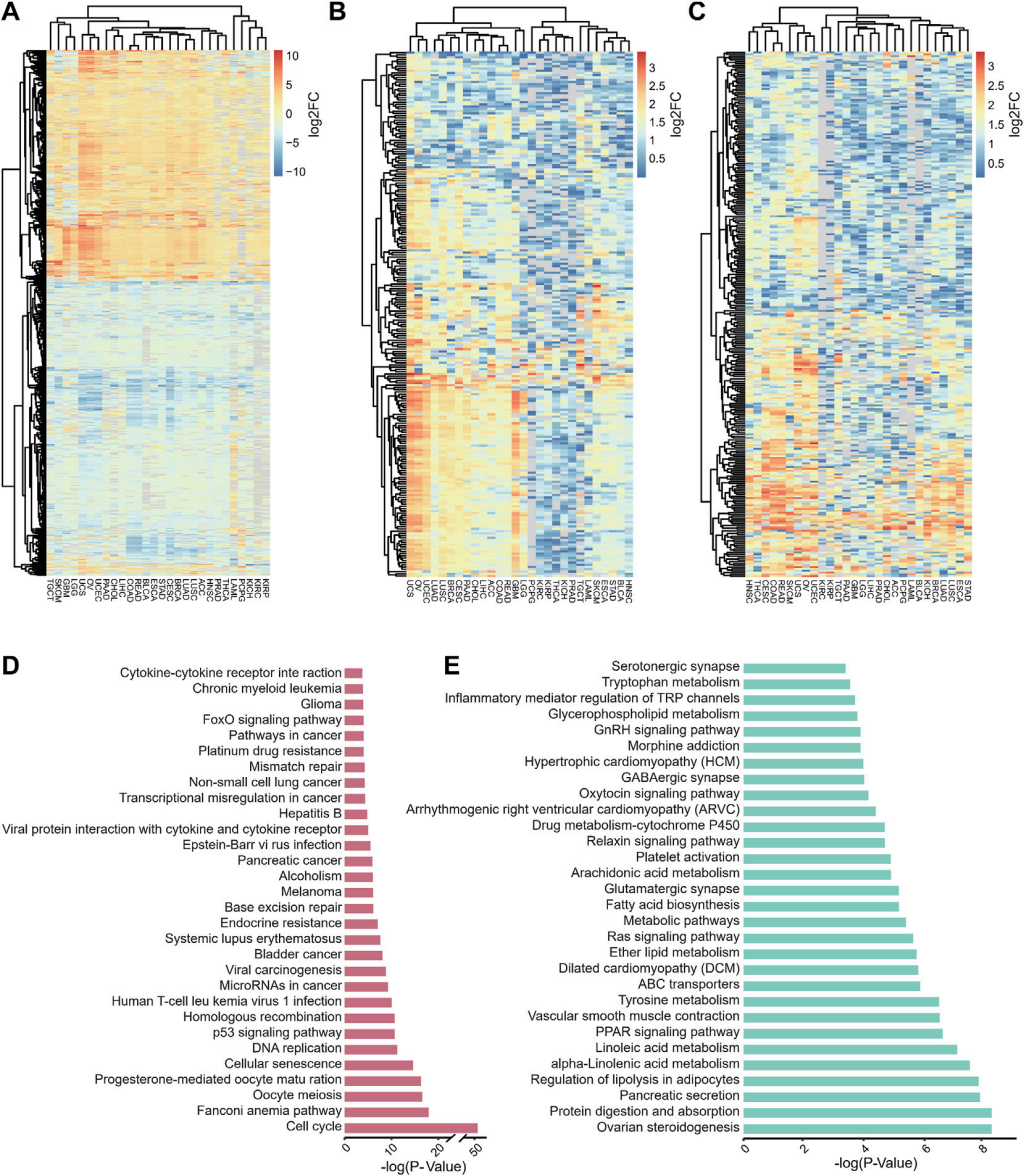
Identified DEGs shared in more than 80% of cancers and pathways significantly associated with these DEGs. **(A–C)** Overview of identified DEGs **(A)**, identical up-regulated **(B)** and down-regulated **(C)** DEGs shared by over 80% of tumors in 28 tumors. **(D–E)** Barplot represents the top 30 enriched pathways of identical up-regulated **(D)** and identical down-regulated **(E)** of DEGs that are shared by over 80% of tumors, analysis was performed using KOBAS 3.0.

We explored similarities in expression using DEGs that were identified in more than 80% of tumors for clustering, and up- and downregulated genes were used separately (Figures 2A–C). Among these studied tumors, some of them with similar expression dysregulation features clustered together, such as COAD and READ were clustered together, probably because they both belonged to colorectal cancer (CRC) (The Lancet Oncology, 2017), while UCS, OV, and UCEC, which are uterine and ovarian cancers, clustered together.

Furthermore, we examined the shared dysregulated pathways of DEGs by KEGG pathway enrichment analysis and found that the shared up-regulated genes among 28 tumors are functionally associated with pathways that are related to oncogenesis and cell cycle (Figure 2D), such as cell cycle, cellular senescence, p53 signaling pathway, human T-cell leukemia virus 1 infection, microRNAs in cancer, transcriptional dysregulation in cancer (Liu et al., 2020; Hirons et al., 2021; Lee and Dutta, 2009). In contrast, the differentially down-regulated expressed genes shared by 28 tumors are mainly involved in nutrient metabolism-related pathways (Figure 2E), such as protein digestion and absorption, regulation of lipolysis in adipocytes, alpha-linolenic acid metabolism, linoleic acid metabolism, tyrosine metabolism, and fatty acid biosynthesis (Burak et al., 2019; Yang and Mottillo, 2020).

### Networks Analysis and Hub Genes Screening

**FIGURE 3:**
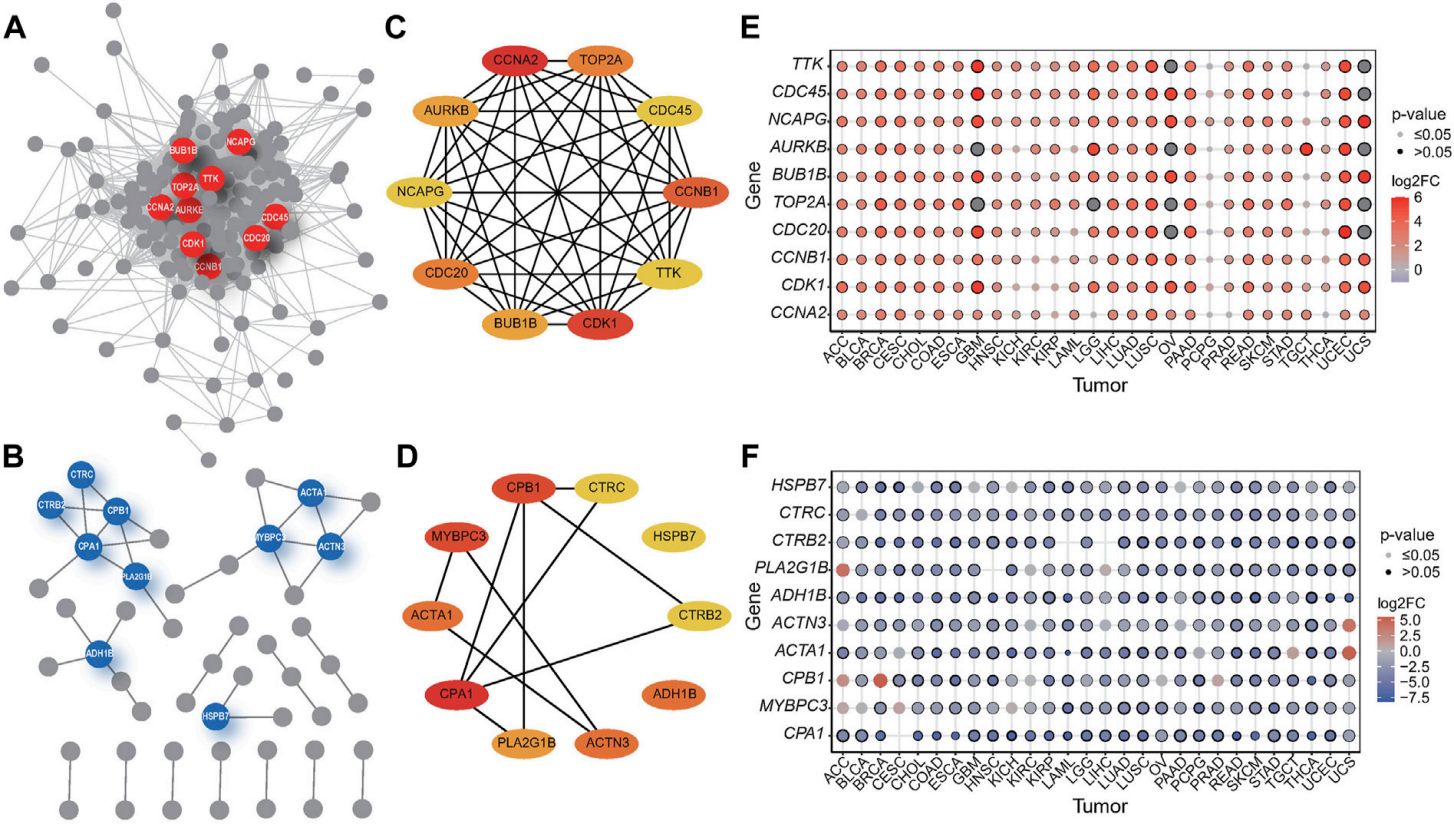
PPI network of the hub genes in tumors and the expression profile of hub genes. **(A–B)** PPI networks of up-regulated **(A)** and down-regulated **(B)** DEGs shared by 28 tumors. The hubs genes were in the center of the network, represented by colored circles. **(C–D)** PPI among top 10 identical up- **(C)** and down-regulated **(D)** DEGs in various cancers. **(E–F)** Gene expression of hub genes in identical up- **(E)** and down-regulated **(F)** DEGs in 28 tumors.

Systematic exploration of the relationships between genes can help to explain the relationship between genotype and phenotype (Kuzmin et al., 2018). We performed protein-protein interaction (PPI) network analysis of the shared DEGs that existed in more than 80% of the tumors using the STRING database and screened the up- and downregulated hub genes using Cytoscape plugin cytoHubba, respectively, based on the amount of connectivity (Figures 3A,B). The highly connected genes in a network are hub genes, and the 10 upregulated hub genes interact much more intensively with each other compared to downregulated hub genes (Figures 3C,D), suggesting that the majority of DEGs in cancers are upregulated, and these hub genes are closely coordinated and interact tremendously to participate in certain oncogenic pathways leading to carcinogenesis.

Next, we mapped the expression levels of the hub genes among these overlapping expression genes in all the studied tumors and presented them as bubble plots (Figures 3E,F) to visualize the differential expression of these genes in specific tumors and para-cancerous tissues. The results showed that *NCAPG* was significantly overexpressed in almost all tumors except TGCT, while *CDC45*, *TTK*, *BUB1B*, and *TOP2A* were overexpressed in all tumors other than PCPG and TGCT.

Non-structural maintenance of chromosomes condensin I complex subunit G (*NCAPG*) is responsible for the condensation and stabilization of chromosomes during mitosis and meiosis (Murphy and Sarge, 2008). The previous study suggests that the overexpression of *NCAPG* is significantly associated with unfavorable survival in diverse human malignancies. And the high expression of *NCAPG* may play an essential role in tumorigenesis and progression (Xiao et al., 2020), serving as a promising molecular target for cancer treatment and prognostic biomarkers for hepatocellular carcinoma (HCC) (Wang et al., 2019b; Xiao et al., 2020).

Cytokinesis cycle protein 45 (*CDC45*) was thought to be associated with tumorigenesis, and its low proteomic levels were associated with poor prognosis in HCC patients, suggesting that *CDC45* may be a novel prognostic marker for HCC (Yang et al., 2021). In addition, knockdown of *CDC45* expression inhibited the proliferation of non-small cell lung cancer (NSCLC) cells *in vitro* and *in vivo* and arrested cells in the G2/M phase of the cell cycle, which could be a novel therapeutic target for NSCLC (Huang et al., 2019). A component of the Spindle Assembly Checkpoint, TTK protein kinase whose inhibition could be a novel therapeutic target for the treatment of triple-negative breast cancer (TNBC) (Maia et al., 2015) and pancreatic ductal adenocarcinoma (PDAC) (Kaistha et al., 2014), is overexpressed in several cancers. BUB1 Mitotic Checkpoint Serine/Threonine Kinase B (BUB1B) is an essential component of the mitotic checkpoint, and its high expression is thought to be associated with the progression and recurrence of several cancers (Dong et al., 2019). Topoisomerase II alpha (TOP2A), highly expressed in various human cancers, is a potential prognostic and predictive marker as well as a therapeutic target in combating HCC (Panvichian et al., 2015; Wang et al., 2022).

### Hub Genes as Potential Diagnostic Markers for Cancers

**FIGURE 4:**
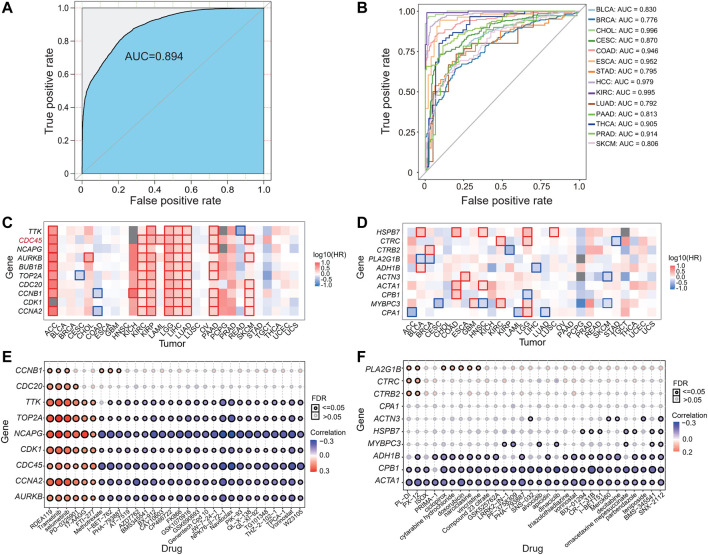
Performance of classification model, prognostic assessment and drug sensitivity evaluation based on hub genes. **(A–B)** Area under the ROC curve (AUC) plots of the training dataset **(A)** and external datasets **(B)**. **(C–D)** Survival analysis of hub genes in identical up- **(C)** and down-regulated **(D)** DEGs in various cancers. **(E–F)** The bubble plots showed the correlations of mRNA expression levels of hub genes with GDSC **(E)** and CTRP **(F)** drug sensitivities.

We used these identified hub genes as features to construct a pan-cancer diagnostic model, and the receiver operating characteristic (ROC) curve illustrated the diagnostic ability and the area under the curve (AUC) showed the performance of a classification model. Based on the Lasso regression, the classifier performed excellently with an AUC value close to 89.4% (Figure 4A) using the top 10 upregulated hub genes (*CCNA2*, *CDK1*, *CCNB1*, *CDC20*, *TOP2A*, *BUB1B*, *AURKB*, *NCAPG*, *CDC45*, and *TTK*). We replicated these hub genes as features for tumor and para-cancerous tissues classification using transcriptomic data from 14 additional datasets for different tumors, all with good accuracy and an AUC of at least 77.6% (Figure 4B). These datasets include BLCA (GSE13507), BRCA (GSE27562), CESC (GSE63514), CHOL (GSE76297), COAD (GSE39582), ESCA (GSE23400), STAD (GSE27342), HCC (GSE14520), KIRC (GSE40435), LUAD (GSE31547), PAAD (GSE62452), PRAD (GSE46602), SKCM (GSE15605), and PATC (GSE33630) (Supplementary Table S3).

### Survival Analysis and Drug Sensitivity Prediction of Hub DEGs

Given that most of the hub genes have been reported to be associated with a variety of tumorigenesis and prognosis, to further explore the role of these genes in the prognosis of all studied tumors, we performed a survival analysis based on these hub genes (Figures 4C,D). We found that 10 up-regulated hub genes were significantly associated with survival in most tumors. For instance, *CDC45* plays a key role in DNA replication was significantly associated with survival in 9 tumors, and *AURKB* was significantly associated with survival in 8 tumors (Figure 4C). We further investigated the expression of *CDC45* in tumors at different stages and found that in all 28 studied tumors, *CDC45* was highly expressed in most tumors compared to normal controls, and its expression level gradually increased with progressive tumor stage (Supplementary Figure S2). Together with the previous finding that *CDC45* affected the survival of most tumors, we speculated that the upregulation of these specific DEGs may have an impact on patients’ survival.

Lastly, to explore the potential of the screened hub genes for clinical application, we calculated the correlation between the hub gene expression and drug sensitivity associated expression profiles from the GSCA database, which merged GDSC and CTRP databases and screened for potential anti-cancer drugs that interacted with it. The result showed that overexpression of these hub genes is positively correlated with the resistance of some drugs, vice verse (Figures 4E,F), implying that these drugs may be able to reduce the expression level of these genes and act as an anti-tumor agent. And some drugs are already used in clinical, such as RDEA119, trametinib, selumetinib, PD-0325901, 17-AAG, and FTI-277 (Figures 4E,F). RDEA119/BAY 869766 is highly potent in inhibiting cell proliferation in several tumor cell lines *in vitro*. It has also shown potent activity in xenograft models of melanoma, colon, and epidermoid carcinoma *in vivo* (Iverson et al., 2009). Trametinib (GSK1120212) is an oral mitogen-activated protein kinase (MEK) inhibitor that is selective for MEK1 and MEK2. It has been approved by the FDA in combination for the treatment of metastatic melanoma with BRAF inhibitors (Zeiser et al., 2018). Selumetinib is a mitogen-activated protein kinase 1 and 2 (MEK1/2) inhibitor, used to treat neurofibromatosis and various cancers. It can also be used as adjuvant therapy for thyroid cancer and the treatment of type 1 neurofibromas (Markham and Keam, 2020). In a word, our analysis and predictions will hopefully be informative for the clinical management of these cancers.

### The Prediction of Immunotherapy Sensitivity

**FIGURE 5:**
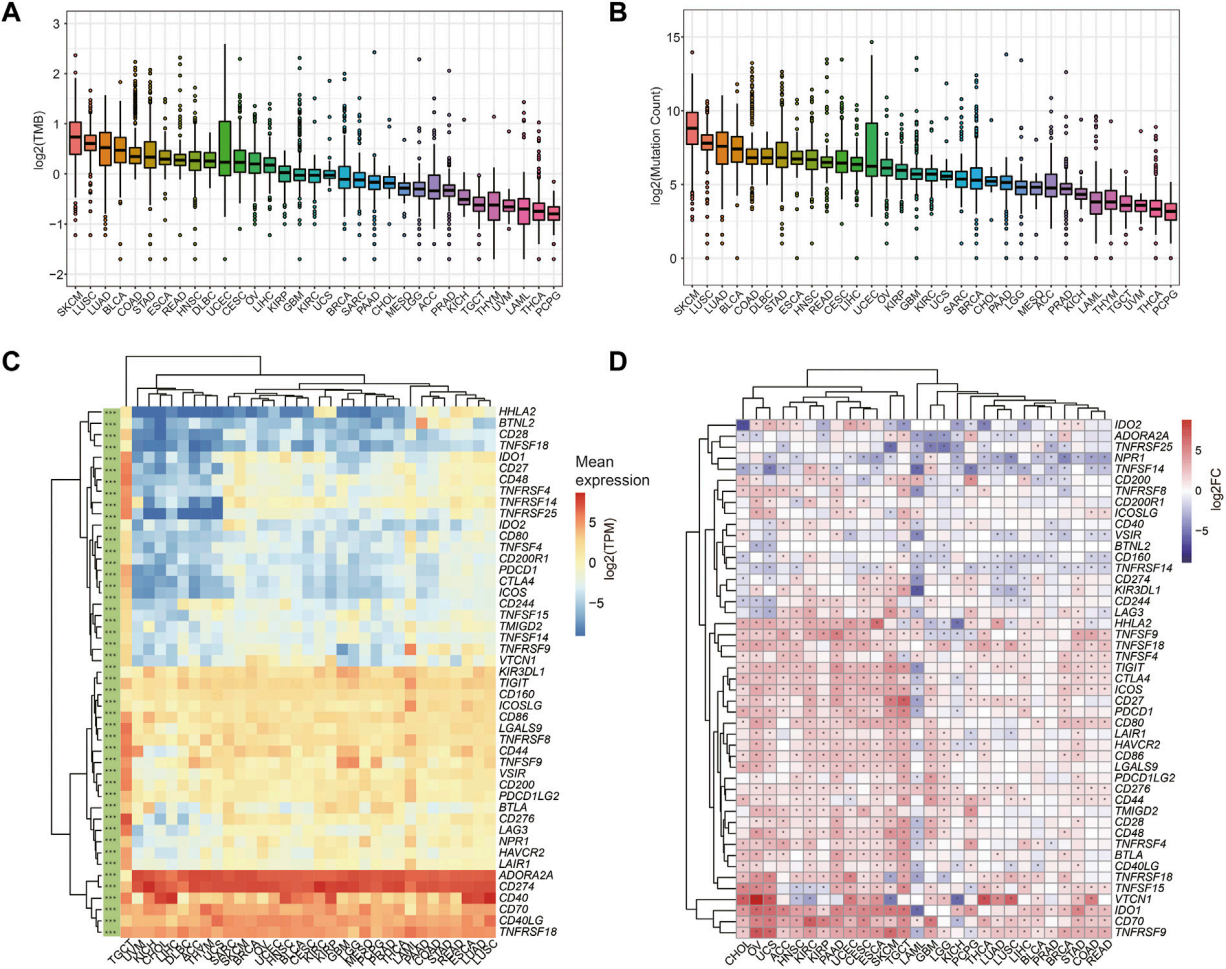
The prediction of immunotherapy sensitivity. **(A–B)** Tumor mutational burden (TMB) **(A)** and mutation count **(B)** across 33 cancer types. **(C)** Gene expression profiling of ICGs in different tumors in the TCGA cohort (* represents that ICGs are significantly differentially expressed in different tumors, Kruskal-Wallis test was used, and *** means *p* < 0.0001). **(D)** Differential expression of ICGs constructed in 28 TCGA cancers with tumor-normal paired samples (* indicates ICGs are differentially expressed in tumor and normal tissues).

Higher TMB and somatic mutation rates were associated with better anti-cancer immune responses (Castle et al., 2019). We calculated the TMB (Figure 5A) and somatic mutation counts (Figure 5B) for all tumor samples based on mutect2 results for 33 tumors in the TCGA. In contrast, patients affected by several tumors with high TMB and mutation counts, such as SKCM, LUSC, and LUAD, may have a higher sensitivity to immunotherapy.

We also collected 47 ICGs reported in the previous study (Huang et al., 2021), and these ICGs are mainly involved in ligand-receptor interactions and have different effects on immune activity, including inhibition, stimulation, or a combination of both. We investigated the expression profiles of ICGs in all tumor tissues of the TCGA cohort (Figure 5C), as well as the differential expression of these genes in 28 tumors and para-cancerous tissues (Figure 5D). Significant differential expression of ICGs was observed in tumors (Figure 5C), and these ICGs had a distinct cancer-specific profile compared to normal controls (Figure 5D). We found that the expression of most ICGs was up-regulated in KIRC, KIRP, ESCA, SKCM, and HNSC compared to para-cancerous tissues, suggesting a potential response to immunotherapy in the corresponding tumors (Zhang et al., 2022).

In addition, we found the ICGs expression was higher in tumors with high TMB and having more mutations such as LUSC, LUAD, ESCA, and STAD (Figure 5C), reinforcing the point that these tumors may have surprising efficacious against prevailing ICIs (Liu et al., 2017; Tung et al., 2021; Xie et al., 2021; Zhang et al., 2021).

## Discussion

Integrating transcriptomic data from a large variety of cancers to study cancer characteristics is an important and valuable direction of research in cancer biology. A substantial number of studies have shown similarities between different cancers, such as key driver mutations (Martincorena and Campbell, 2015), immune (Desrichard et al., 2016), and microbial signatures (Poore et al., 2020), suggesting the possibility of common features of different cancers for tumor diagnosis and clinical recommendations. However, cancer cell heterogeneity is a challenging concept in cancer biology. Therefore, we herein sought to find potential biomarkers by selecting dysregulated genes in most types of cancer from a large cohort and validating these biomarkers in additional independent datasets to reduce the impact of heterogeneity across cancers.

In this study, we analyzed a large amount of transcriptomic data from public databases of tumor and para-cancerous tissues and obtained DEGs that were differentially expressed in most of the studied tumors compared to para-cancerous tissues, some of which have been reported can be used as diagnostic or prognostic markers for specific cancer (Kaistha et al., 2014; Maia et al., 2015; Wang et al., 2019b; Xu et al., 2019; Xiao et al., 2020; Zhao et al., 2021; Luo et al., 2022) and partly as biomarkers for pan-cancer diagnosis (Dong et al., 2019; Jiang et al., 2021; Pan et al., 2021). We performed PPI network analysis on genes that differentially expressed in more than 80% of the studied cancers and obtained 20 up- and downregulated hub genes. We further explored the possibility of using the ten upregulated hub genes as features to distinguish tumors from para-cancerous tissues, which achieved high accuracy and sensitivity. Furthermore, we found that some hub genes have the potential for the prognostic assessment of cancer patients. Additionally, we examined the drug sensitivity of some broad-spectrum anti-cancer drugs based on 20 hub genes and obtained a handful of drugs such as RDEA119, trametinib, and selumetinib possibly exert anti-tumor effects in pan-cancers with corresponding DEGs.

Finally, we found that some tumors such as SKCM, LUSC, LUAD, KIRC, KIRP, ESCA, HNSC, and STAD might be more suitable for immunotherapy by comparing the TMB, mutation count levels as well as the expression levels of ICGs in different tumors. Interestingly, from the rank of TMB and somatic mutation counts, TGCT may be unsuitable for immunotherapy. However, regarding the expression of ICGs, TGCT is possibly sensitive to ICIs, which needs to be explored in further studies (Kalavska et al., 2020). In addition, ICGs such as *CD276* and *CD70* are upregulated in most tumors, and gene expression profiles show that *CD276* is highly expressed in tumors such as SKCM, HNSC, ESCA, and LUSC, while *CD70* is highly expressed in the majority of tumors. High expression of the *CD276* gene is thought to be associated with the development and metastasis of several cancers (Yuan et al., 2011; Boland et al., 2013; Mao et al., 2015), and *CD70* is involved in the survival of tumor cells and regulatory T cells through interaction with its ligand *CD27* (Jacobs et al., 2015). A previous study showed that tandem CAR-T cells targeting CD70 and CD276 exhibited potent preclinical activity against a variety of solid tumors (Yang et al., 2020), suggesting that these two genes could be candidate targets for immunotherapy. However, this may be influenced by resistance to anti-cancer immunotherapy, the development of which involves complex and diverse factors. Thus pre-treatment testing of oncology patients and assessment of possible resistance development would be beneficial in guiding the choice of anti-cancer immunotherapy. In addition, combinations of ICIs, or combinations of strategies (cancer immunotherapies with targeted mutagenic drugs) may be considered to overcome the resistance to anti-cancer immunotherapy and improve efficacy (Sharma et al., 2017).

Despite the compelling results found in our study, there are still some limitations to be noted. First, cancer is a highly heterogeneous disease with great variation between tumors. Although the hub genes we identified hereby can predict patient survival to some extent, the survival of specific cancer patients is influenced by many factors. Except for these genetic background factors, whether the patient can be diagnosed at an early stage, the status of mainstream treatment modalities, and the accessibility of new treatments (especially immunotherapy) will lead to differences in survival outcomes.

Second, although our work suggests several biomarkers that may be useful for pan-cancer diagnosis and broad-spectrum anti-cancer drug selection, it should be noted that the present study is a retrospective study without validation in independent wet-lab laboratory experiments, and the limited sample size for some cancers could potentially lead to inaccurate or false-positive results. Besides, ICG expression was quantified at the transcriptomic level, while proteins such as mutation-derived neoantigens are the components directly involved in tumor immunity, and the protein expression does not exactly match the RNA expression (Dybas et al., 2019), so the performance of RNA expression-based biomarker may be influenced by the inconsistency between the RNA expression and protein expression. We believe that further persuasive wet-lab experiments and clinical studies are still needed to validate these biomarkers and confirm their specificity and sensitivity.

Third, we mainly explored the DEGs between tumor and para-cancerous tissues but did not address the discrepancies among different tumors. Future studies on specific markers for different tumors can be considered, which are expected to distinguish metastatic tumors that cannot be determined from the primary lesions and achieve early diagnosis and precision medicine for indistinguishable tumors.

In summary, the hub genes identified in this study may serve as biomarkers to construct a pan-cancer diagnostic model that could effectively distinguish tumors from para-cancerous tissues and contribute to drug selection and development. Our findings provide new clues for pan-cancer classification in complex cancer biology and facilitate early diagnosis and precise treatment of cancer.

## Data Availability

The original contributions presented in the study are included in the article/Supplementary Material, further inquiries can be directed to the corresponding authors.
