# Supplementary figures and images for: Identification of Potential Biomarkers for Pan-Cancer Diagnosis and Prognosis Through the Integration of Large-Scale Transcriptomic Data
Source: Front Pharmacol. 2022 May 23;13:870660. doi: 10.3389/fphar.2022.870660 (PMC9169228; doi:10.3389/fphar.2022.870660)

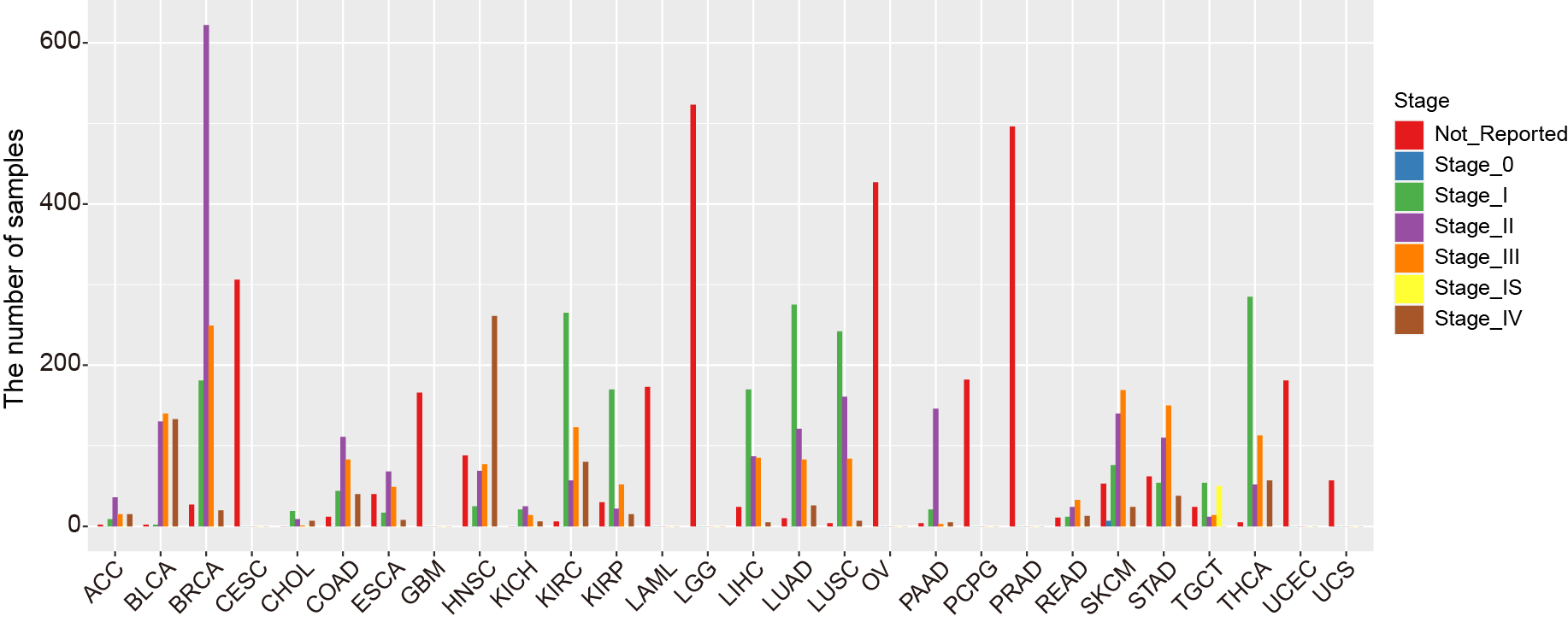

Supplement: Supplementary file 4 [file Image1.PNG]

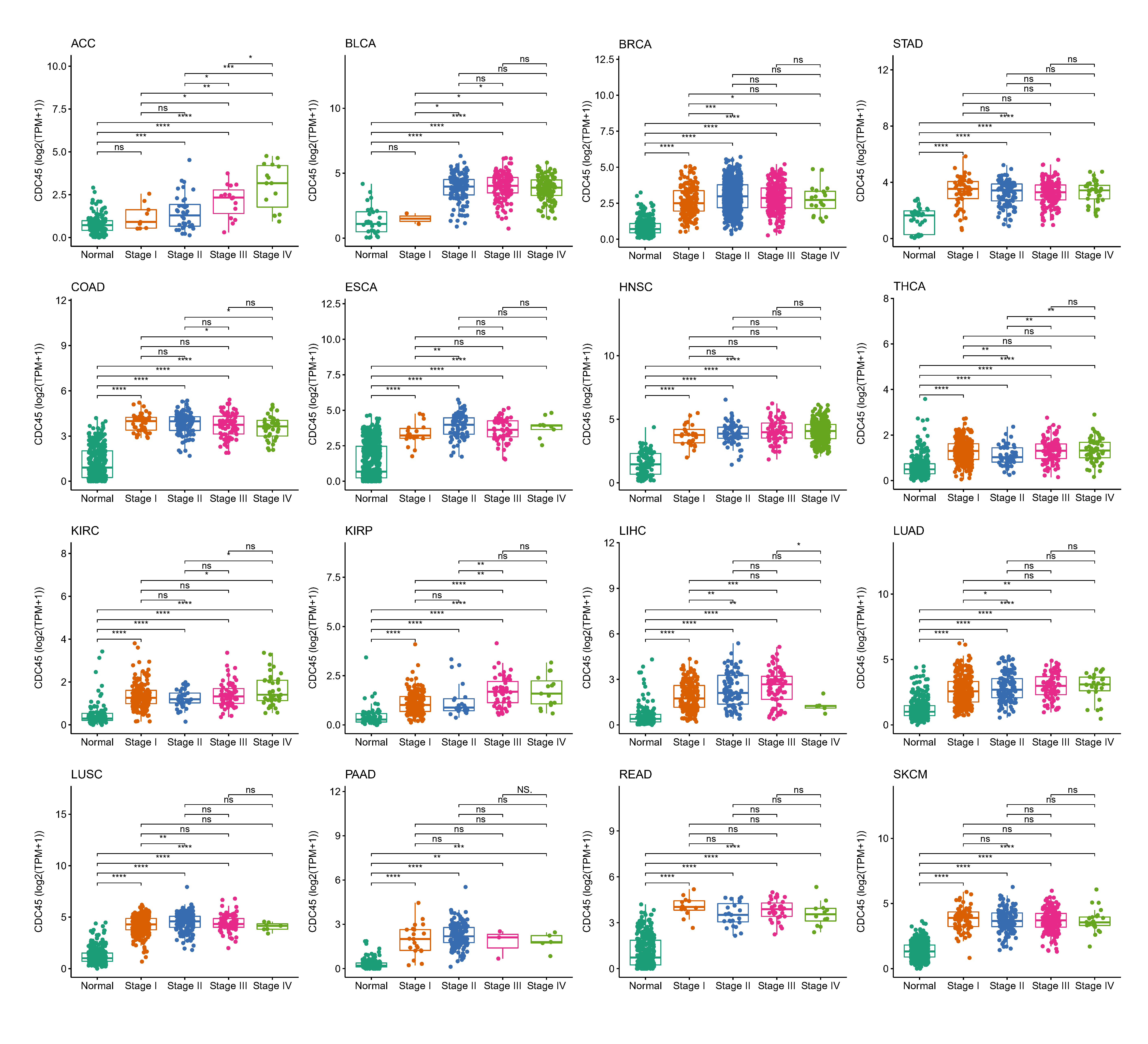

Supplement: Supplementary file 5 [file Image2.TIFF]
